# Diagnostic performance of lung ultrasound compared to thoracic radiography in non-traumatized dogs and cats with respiratory distress in an emergency setting

**DOI:** 10.3389/fvets.2026.1790755

**Published:** 2026-04-15

**Authors:** Doris Bittenecker, Kaye Mohr, Florian Sänger, Rene Dörfelt, Katarzyna Kraszewska, Wolfgang Henninger, Gerhard Wess

**Affiliations:** 1 LMU Small Animal Clinic, Centre for Clinical Veterinary Medicine, Faculty of Veterinary Medicine, Ludwig-Maximilians-University, Munich, Germany; 2 Vetcardia Veterinary Clinic, Warsaw, Poland; 3 Diagnostic Center for Small Animals, Vienna, Austria

**Keywords:** canine, dyspnea, emergency diagnostic imaging, feline, point-of-care ultrasound, thoracic radiographs

## Abstract

**Introduction:**

Respiratory distress is a medical emergency requiring rapid diagnostic decision-making. This prospective observational study evaluated the efficiency and diagnostic performance of lung ultrasound (LUS) and thoracic radiography (TXR) in dogs and cats presenting with tachypnea. Imaging-based suspected diagnoses were compared with definitive diagnoses established by specialists using additional diagnostic procedures. The influence of observer experience on diagnostic accuracy was assessed.

**Methods:**

Client-owned animals with suspected intrathoracic causes of respiratory distress underwent both LUS and TXR within 24 h of presentation, with a maximum interval of 1 h between examinations. Imaging was performed according to standardized protocols. Based on imaging findings, the suspected cause of breathing difficulties was categorized as: no detectable intrathoracic cause, cardiac-related respiratory distress, pulmonary disease, neoplasia, or pleural effusion of unknown etiology. Following stabilization, patients were referred to specialists for establishment of the definitive diagnosis. Concordance between suspected and definitive diagnoses was analyzed separately for each modality to assess diagnostic performance. Selected cases were re-evaluated in a blinded fashion by observers with three different levels of experience.

**Results:**

A total of 144 animals (83 dogs and 61 cats) with acute respiratory distress were included. The suspected diagnosis based on LUS was concordant with the definitive diagnosis in 80.6% of cases, while TXR achieved 89.1%. Both modalities were correct in 77.5% of cases, and at least one modality was correct in 92.2%. Diagnostic accuracy of TXR was statistically significantly higher than that of LUS (absolute difference 8.5%). In dogs, the presence of a heart murmur was strongly associated with a cardiac cause of respiratory distress (odds ratio 10.9), whereas this association was not statistically significant in cats. In the blinded re-evaluation, TXR demonstrated higher interobserver agreement (Fleiss’ kappa = 0.69). The highest agreement was achieved by a less experienced observer, with a Cohen’s kappa of 0.8 for TXR.

**Conclusion:**

Both LUS and TXR are valuable imaging modalities for initial classification of respiratory distress, each offering specific advantages depending on the underlying pathology. In most cases, these techniques provide sufficient information to guide initial therapeutic decisions; however, a multimodal approach is recommended for complex presentations.

## Introduction

In patients with respiratory distress, rapid identification of the underlying cause, minimal handling, and prompt initiation of treatment are crucial for achieving a favorable outcome. This is particularly important in cats which are highly sensitive to stress and respiratory compromise. Forced positioning may worsen respiratory compromise and, in severe cases, precipitate respiratory or cardiac arrest. To determine the underlying cause of increased respiratory effort, diagnostic testing is essential, as both the range and severity of clinical signs can vary widely. Physical examination may offer hints but rarely leads to a diagnosis. Thoracic radiographs (TXR) have traditionally been the diagnostic imaging modality of choice for animals with intrathoracic respiratory diseases. However, the emergence of lung ultrasound (LUS) has provided a less stressful alternative for both dogs and cats, allowing for more rapid and gentle assessment of thoracic pathology. Thoracic ultrasound is becoming increasingly popular in small animal medicine, aided by the availability of various examination protocols tailored to different clinical settings. Initially, it was primarily used for trauma patients in the form of the thoracic-focused assessment with sonography for trauma (T-FAST) protocol (1). Over the last few years, a lot of work has been published, as the application is expanding to gather more knowledge and improve patient care. There is a study comparing LUS with TXR (2), another one tested its prediction value together with biomarkers (3) and also one that compared LUS diagnostic accuracy with computed tomography (4). The shown advantages of this imaging modality include its relative affordability, portability of modern ultrasound devices, absence of radiation exposure, ease of use with relatively little training and duration of the procedure. Additionally, animals can often be examined in a position of their own choice, which helps reduce stress and allows for the administration of supplemental oxygen during the examination if needed.

The Veterinary bedside lung ultrasound examination (Vet BLUE®), as described by (5), is a rapid point-of-care LUS, used as a first line screening diagnostic to differentiate respiratory versus non-respiratory causes and discriminate between the possibly affected organ systems. At present, detailed descriptions are available outlining the procedure, along with its advantages and limitations (5). Further, studies have demonstrated the utility of the Vet BLUE® protocol in detecting cardiogenic pulmonary edema (CPE), monitoring its resolution and diagnosing other thoracic diseases, for example, pneumonia, in both dogs and cats (6–9).

The primary objective of this study was to evaluate the accuracy of a modified Vet BLUE® protocol in identifying the primary intrathoracic pathology responsible for respiratory distress in dogs and cats, and to compare its performance with that of TXR. Additionally, the study aimed to determine whether one imaging modality should be preferred, and whether certain clinical parameters increase the likelihood of reaching a certain diagnosis. As a secondary objective, a subset of patients underwent blinded, repeated evaluations of each imaging modality by observers with three different levels of expertise: a recently graduated veterinarian, a resident in emergency and critical care and a diplomate in either diagnostic imaging or ECC, as well as a veterinarian with over 7 years’ experience in performing LUS.

We hypothesized that LUS would demonstrate diagnostic performance comparable to TXR in identifying the primary intrathoracic pathology responsible for respiratory distress in an emergency setting, with potential advantages in selected diagnostic categories. For the blinded re-evaluation component, we anticipated that TXR would show higher reproducibility and interpretative consistency on repeated assessments.

## Materials and methods

This prospective observational study included dogs and cats which were presented at the emergency service of the Small Animal Clinic, Faculty of Veterinary Medicine, Ludwig-Maximilians-University, Munich, over a 16-months’ period (March 2021 to June 2022). Animals showing any kind of breathing difficulties, such as tachypnea, polypnea, labored breathing, increased work of breathing or increased respiratory effort were considered for inclusion if the emergency veterinarian initially suspected an intra-thoracic cause of respiratory distress. Exclusion criteria were systemic and other causes for increased respiratory effort, such as pain, trauma history, hyperthermia, anemia, severe metabolic acidosis, or upper airway diseases. The study was approved by the ethics committee of the faculty of veterinary medicine, Ludwig-Maximilians-University, Munich (protocol no. 354–19-03-2023), and the owner’s consent for inclusion in the study was obtained for each animal.

Patients were stabilized with oxygen, and sedation was allowed at the discretion of the emergency clinician. In 119 patients no sedation was given. In the remaining 25 animals (8 dogs and 17 cats) medication was administered, 23-times butorphanol with a dosage of 0.2 mg/kg either intramuscularly or intravenously. In addition to butorphanol, either midazolam (0.2 mg/kg) or alfaxalone (1 mg/kg) was added in one cat each to be able to perform both examinations and a thoracocentesis. Due to the small number of sedated patients, no further analysis was performed with this subgroup. A full physical examination was performed either on arrival or at a later timepoint in instable patients.

No additional study-specific training was provided prior to the data collection period. Lung ultrasound and thoracic radiography are part of routine emergency diagnostics in our clinic, and emergency veterinarians receive supervised onboarding training in both modalities upon starting their position. Both diagnostic imaging modalities were conducted within the first 24 h after presentation and animals were only included if their condition allowed undergoing TXR and LUS within 1 h. Ideally, no medication was administered prior imaging, but this could only be ensured in first opinion cases. Any prior therapy was tolerated so that referred cases or patients on long-term medication could be included too. Preferably, TXR was performed before LUS to avoid potential interference of residual moisture on the fur with radiographic image acquisition; however, the order was allowed to vary depending on the patient’s clinical condition. Only patients clinically stable enough to tolerate at least two radiographic projections were included, in order to ensure sufficient diagnostic quality and comparability between cases. For both imaging modalities predefined assessment protocols were created to assure good comparability. On each form certain key points for analyzing the images were given, and the same protocol was used by the emergency veterinarian as well as for blinded re-evaluation.

The TXR were performed in lateral and ventrodorsal or dorsoventral recumbency, so positioning was chosen by the emergency veterinarian depending on the patient’s tolerance and condition. An attempt was made to achieve the best possible inspiration. All images were taken with the same X-ray device (Fujifilm FDR Smart X) using predefined weight-based exposure protocols. The evaluation form included assessment of the diaphragm, presence of free fluid or air, vertebral heart score considering breed specific reference ranges in dogs, vena cava caudalis, pulmonary vessels and the characteristics and distribution of lung patterns.

**Figure 1:**
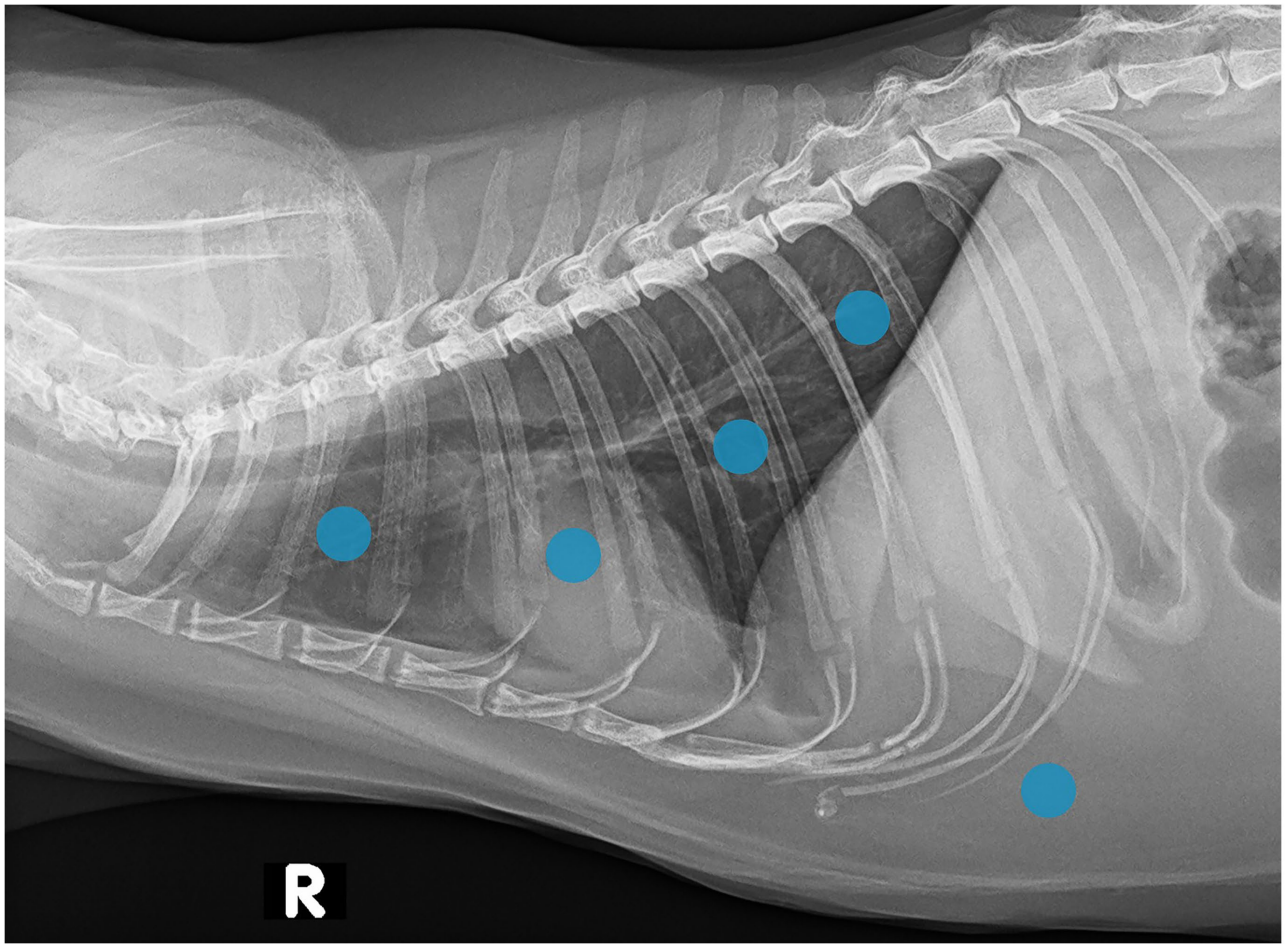
Lateral thoracic radiograph of a cat showing a schematic representation of the acoustic windows used for performing LUS (four each hemithorax with an additional xiphoidal view). As described in the Vet BLUE® protocol, slight deviations were permitted in order to achieve the best possible image quality.

A modified Vet BLUE® was performed, including four acoustic windows on each hemithorax as well as a xiphoidal view. The additional xiphoidal acoustic window was included to improve detection of pleural and pericardial effusion. This approach may reduce lung air interference and enhance visualization of fluid accumulation in the ventral thoracic cavity. A previous study has demonstrated a high success rate (83%) in identifying pericardial effusion using this view (10). The acoustic windows of the modified protocol are illustrated in Figure 1. If the emergency vet deemed it important and was able to perform it, an individual brief assessment of the heart was done as well. All LUS examinations were carried out by use of a single portable ultrasonographic machine (GE Logiq V2, GE medical systems) with a microconvex probe (8C-RS; 6,0–10,0 MHz), starting with standardized settings for either small or large animals with a frequency of 10 MHz. The depth was initially set at 4–7 cm for the thoracic views, with the pleural line being in the upper third of the screen, and was increased as much as necessary for the xiphoidal view as well as for the cardiac site. The focus point was initially set at about 2.5 cm, and was adjusted if necessary. Patients were allowed to stand, sit or lie in sternal position, according to their preference. The fur was parted, and alcohol was applied to ensure optimal contact between the transducer and the skin. The probe was positioned horizontally, with slight cranial, caudal, dorsal, or ventral angulation permitted to optimize image acquisition. By the end of the examination, a minimum of nine 3-s cine loops were recorded and stored for immediate analysis and later reassessment. The evaluation form included assessment for each window: glide sign, B-lines and their quantity, shred sign, tissue sign and effusion, and any findings of the heart when it was screened. For each evaluated ultrasound artefact, we cite one study exemplary: B-lines were defined as hyperechoic vertical lines, arising from the pulmonary surface, moving with respiration, and extending to the far field of the ultrasound screen (11). The shred and tissue sign are both lung consolidations, the first being less severe: A shred sign is seen when there is fluid in the alveolar space, but there are still air dynamic or static bronchograms visible, depicting a certain degree of aeration. It presents with hypoechoic appearance and the margins are irregular. This is in contrast to the tissue sign, where the margins usually are smooth, the echogenicity can vary and it has a more parenchymal appearance (12).

Following the initial clinical and diagnostic examinations, the primary veterinarian – who was not blinded to any of the findings or images in order to ensure optimal treatment and patient care – formulated a tentative diagnosis and initiated treatment. For the purpose of simplified analysis, patients were classified into the following diagnostic categories: no detectable intrathoracic cause, cardiac-related respiratory distress, increased respiratory effort due to lung disease (including lower airway and pulmonary parenchymal diseases, excluding CPE), neoplasia, and pleural effusion of unknown etiology. Cases for which no definitive diagnosis could be established were excluded from further analysis.

Following stabilization, each patient was referred to the appropriate board-certified specialist (e.g., cardiology, internal medicine), depending on the suspected affected organ system. The definitive diagnosis was established by the responsible specialist based on the complete clinical evaluation and all available diagnostic findings. Additional diagnostic procedures (such as laboratory testing, echocardiography, bronchoscopy, advanced imaging, or post-mortem examination) were performed as deemed clinically necessary on a case-by-case basis. No standardized diagnostic protocol was imposed beyond the initial imaging procedures.

Additionally, data from the initial physical examination were collected to assess potential coherence. These parameters included general condition, mucous membrane color, capillary refill time, heart rate, presence of a heart murmur, pulse quality, respiratory rate and pattern, lung auscultation findings, hydration status, and body temperature.

Blinded re-evaluation to test interobserver agreement and influence of expertise was performed with anonymized images in DICOM format in 30 randomly chosen patients, and the observers were blinded to the findings of the other imaging modality as well as the final diagnosis. Only a short signalment was given, as well as the most important clinical findings and treatments performed prior to imaging. The analysis was performed at three different levels of experience for both modalities separately: veterinarian with approximately 2 years of clinical experience in emergency practice,

where TXR and LUS are part of routine diagnostics and an ECC resident with 5 years of experience as a veterinarian. Those two evaluated both modalities, with cases set in a different order and about 3 weeks in between, to avoid bias of remembering details from some patients. Further, a diplomate in diagnostic imaging re-evaluated the TXR, and an ECC diplomate and an experienced veterinarian with more than 7 years working in the field of LUS re-evaluated the LUS examinations.

### Statistical analysis

Statistical analysis was performed by use of a commercially available statistical software (IBM SPSS 29.0, NY, United States). Normality was tested using the Shapiro–Wilk test, and data were given as median and range as none of the data were normally distributed. The Chi-square test of association was performed to assess the relationship of LUS, respectively, TXR with the definitive diagnosis.

For paired comparison of diagnostic accuracy between LUS and TXR, McNemar’s test was applied using dichotomous paired outcomes (correct vs. incorrect relative to the definitive diagnosis), both overall and within each definitive diagnostic category. This test was also conducted to evaluate if there was a significant difference between the overall performance of the two modalities and to assess the proportional difference. For all different categories, sensitivity and specificity was calculated (suppl. data). Logistical regression analysis or a Chi-square test was used to assess the impact of additional variables (i.e., heart murmur and heart abnormalities on ultrasound) with separated analysis for dogs and cats. Interobserver agreement was calculated using the Fleiss’ or Cohen’s kappa. The significance level was set at *p* < 0.05.

## Results

### Patient population and demographics

One hundred forty-four client-owned pets (83 dogs and 61 cats) were enrolled in the study. The most represented cat breeds were Domestic Shorthair (*n* = 38) and British Shorthair (*n* = 5), further 10 additional breeds with ≤ 4 cats each were also included. In dogs, the three most common breeds were Chihuahua (*n* = 16), mixed breed (*n* = 15) and Yorkshire Terrier (*n* = 6); 34 additional breeds were represented by ≤4 dogs each. The median (range) age of the whole population was 9.8 (0.1–19.6) years. The median (range) body weight for cats was 4.3 (1.0–13.8) kg, and for dogs it was 8.3 (1.3–64.0) kg. The median (range) time for inclusion was 1.25 (0–24) h, with 71.5% of animals being included within the first 3 h after arrival.

**Figure 2:**
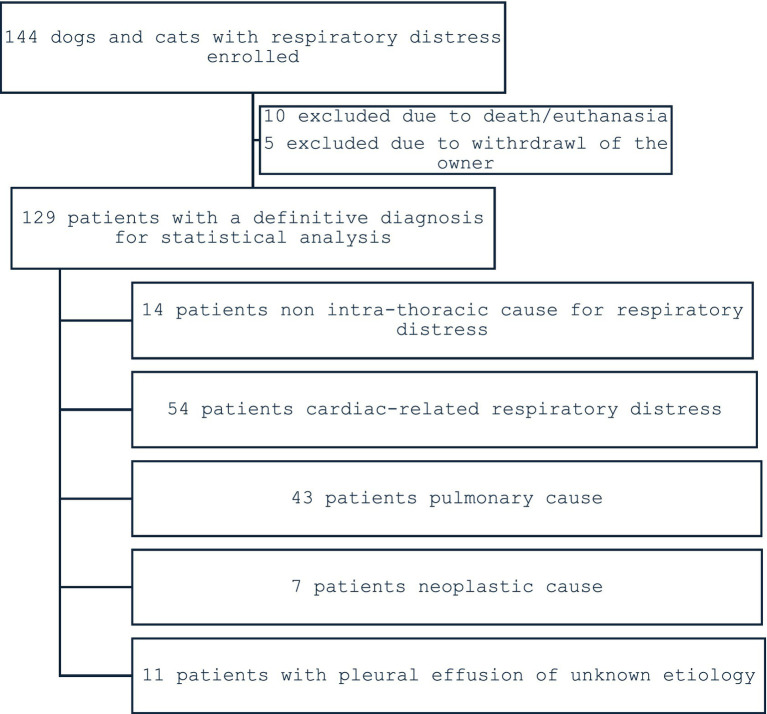
Flow chart of the case distribution among the categories.

Fifteen patients were excluded, because the animals died or were euthanized before completion of specialist evaluation (*n* = 10), or their owners withdrew from further diagnostic workup (*n* = 5). The excluded animals consisted of 10 dogs and 5 cats (age range 4–17 years). Due to incomplete diagnostic evaluation and absence of post-mortem confirmation, further classification was not possible. Further analysis was performed with the remaining 129 animals (89.6%; 73 dogs and 56 cats) (Figure 2).

### Concordance of diagnosis

At first, the overall association was evaluated: The tentative diagnosis based on the LUS was consistent with the definitive one in 104 cases (80.6%), whereas the TXR suspected diagnosis matched the definitive one in 115 of them (89.1%). Both modalities were consistent with the definitive diagnosis in 100 cases (77.5%), and one of the two modalities was correct in 119 cases (92.2%). In four cases (3.1%) the correct categorization was only achieved with LUS, whereas in 15 cases (11.6%) the correct suspected category was only obtained with TXR. Furthermore, the association was evaluated separately for each of the five categories (Tables 1, 2). With the Chi-square test both modalities reached statistical significance for association with *p* < 0.001. TXR showed a slightly higher diagnostic performance and sensitivity across all categories, except for pleural effusion of unknown etiology. McNemar’s test did not reveal statistically significant differences between LUS and TXR within individual diagnostic categories. In the overall paired comparison, 15 cases were correctly classified by TXR but not by LUS, whereas 4 cases were correctly classified by LUS but not by TXR (McNemar’s test, *p* = 0.019).

**Table 1:** Association of suspected affected organ system causing respiratory distress comparing lung ultrasound (LUS) with the definitive diagnosis in the entire patient population (*n* = 129).

	Definitive diagnosis						
		Not intra-thoracic	Cardiac-related respiratory distress	Pulmonary cause	Neoplasia	Pleural effusion of unknown etiology	Total
Suspected affected organ system LUS	Not intra-thoracic	12 85.7%	1 1.9%	8 18.6%	0%	0%	21 16.3%
	Cardiac-related respiratory distress	0%	47 87%	4 9.3%	1 14.3%	0%	52 40.3%
	Pulmonary cause	2 14.3%	3 5.6%	30 69.8%	0%	0%	35 27.1%
	Neoplasia	0%	0%	1 2.3%	4 57.1%	0%	5 3.9%
	Pleural effusion of unknown etiology	0%	3 5.6%	0%	2 28.6%	11 100%	16 12.4%
	Total	14 100%	54 100%	43 100%	7 100%	11 100%	129 100%

**Table 2:** Association of suspected affected organ system causing respiratory distress comparing thoracic radiographs (TXR) with the definitive diagnosis in the entire patient population (*n* = 129).

	Definitive diagnosis						
		Not intra-thoracic	Cardiac-related respiratory distress	Pulmonary cause	Neoplasia	Pleural effusion of unknown etiology	Total
Suspected affected organ system TXR	Not intra-thoracic	13 92.9%	0%	3 7.0%	0%	0%	16 12.4%
	Cardiac-related respiratory distress	0%	50 92.6%	2 4.7%	1 14.3%	1 9.1%	54 41.9%
	Pulmonary cause	1 7.1%	1 1.9%	38 88.4%	0%	1 9.1%	41 31.8%
	Neoplasia	0%	1 1.9%	0%	5 71.4%	0%	6 4.7%
	Pleural effusion of unknown etiology	0%	2 3.7%	0%	1 14.3%	9 81.8%	12 9.3%
	Total	14 100%	54 100%	43 100%	7 100%	11 100%	129 100%

A logistic regression analysis was performed to assess the odds ratio of a heart murmur, leading to the definitive diagnosis of a heart disease being the cause for the breathing difficulties. Auscultation at first presentation was feasible for the emergency veterinarian in 68 of 73 dogs with a definitive diagnosis (93.2%) and in 49 of 56 cats (87.5%). Reasons why auscultation of the heart was not or only partially possible were severely increased respiratory effort, panting or intensified lung sounds. A cardiac cause being the definitive diagnosis for labored breathing in auscultated animals was found in 25 dogs and 23 cats. Four dogs with the heart being the main reason for respiratory distress did not have a heart murmur, as well as 16 cats within this category. When considering only the dogs in which auscultation was feasible, the presence of a heart murmur in a dog made a heart disease being the cause of respiratory distress significantly more likely with an odds ratio of 10.9 (*p* < 0.001) (Table 3). In cats, it did not reach statistical significance (*p* = 0.215), with an odds ratio of 2.4 (Table 4). Other parameters of the physical examination failed to reach the significance level and thus are not mentioned here.

**Table 3:** Cross table on the effect of a heart murmur auscultated by the emergency veterinarian in dogs leading to the definitive diagnosis of a heart disease being the cause for respiratory distress.

		Definitive diagnosis cardiac-related respiratory distress		
		No	Yes	Total
Heart murmur	No	29 67.4%	4 16.0%	33 48.5%
	Yes	14 32.6%	21 84.0%	35 51.5%
	Total	43 100%	25 100%	68 100%

**Table 4:** Cross table on the effect of a heart murmur auscultated by the emergency veterinarian in cats leading to the definitive diagnosis of a heart disease being the cause for respiratory distress.

		Definitive diagnosis cardiac-related respiratory distress		
		No	Yes	Total
Heart murmur	No	22 84.6%	16 69.6%	38 77.6%
	Yes	4 15.4%	7 30.4%	11 22.4%
	Total	26 100%	23 100%	49 100%

Furthermore, it was evaluated if an individual assessment of the heart was performed during the ultrasound at presentation, and if that had an impact on the diagnosis. In 27/73 dogs (37%) the emergency vet considered it necessary, or it was clinically feasible to evaluate the heart during LUS. When an abnormality was detected, a cardiac cause for the respiratory distress was statistically significantly more likely (*p* < 0.001) (Table 5). A logistic regression analysis was not possible, as there was no dog with an unremarkable heart on ultrasound, but the definitive diagnosis of a heart disease being responsible for the respiratory symptoms. In cats, a logistic regression analysis was performed to evaluate the same effect. In 22/56 cats (39.3%) a cardiac evaluation during LUS was feasible, due to the same reasons as in dogs. If heart abnormalities were found, a heart disease being the cause of respiratory distress was 45 times more likely, which was statistically significant (*p* = 0.004) (Table 6).

**Table 5:** Cross table used to perform a Chi-square test of cardiac abnormalities detected during the ultrasound in dogs and the effect of the finding on the definitive diagnosis of a cardiac cause for respiratory distress.

		Definitive diagnosis cardiac-related respiratory distress		
		No	Yes	Total
Heart abnormality with ultrasound—dogs	No	7 63.6%	0 0%	7 25.9%
	Yes	4 36.4%	16 100%	20 74.1%
	Total	11 100%	16 100%	27 100%

**Table 6:** Cross table used to perform a logistic regression analysis of cardiac abnormalities detected during the ultrasound in cats and the effect of the finding on the definitive diagnosis of a cardiac cause for respiratory distress.

		Definitive diagnosis cardiac-related respiratory distress		
		No	Yes	Total
Heart abnormality with ultrasound—cats	No	9 81.8%	1 9.1%	10 45.5%
	Yes	2 18.2%	10 90.9%	12 54.5%
	Total	11 100%	11 100%	22 100%

For the blinded re-evaluation, 30 randomly selected cases were independently reassessed using anonymized DICOM files. Both imaging modalities (LUS and TXR) were evaluated separately by observers representing three different levels of expertise. Interobserver agreement was assessed using Fleiss’ kappa for overall agreement and Cohen’s kappa for pairwise agreement. Results are summarized in Table 7. Overall interobserver agreement was moderate to substantial for both modalities (Fleiss’ kappa: LUS 0.63; TXR 0.69), with slightly higher agreement observed for TXR.

**Table 7:** Kappa values of the blinded re-evaluation.

	Fleiss’ kappa	Rater	Cohen’s kappa
TXR	0.69	Emergency vet	0.80
		ECC resident	0.73
		Diagnostic imaging diplomate	0.75
LUS	0.63	Emergency vet	0.63
		ECC resident	0.69
		ECC diplomate	0.61
		LUS expert	0.61

TXR, thoracic radiographs; LUS, lung ultrasound.

## Discussion

The results of the present study provide insight into the diagnostic performance of LUS and TXR in non-traumatized small animal patients presenting with respiratory distress in an emergency room setting. Even when a definitive diagnosis cannot be established through initial diagnostics, categorization of the suspected pathology helps guide appropriate therapy and leads to rapid symptom improvement. This approach also aids in assessing prognosis, determining the need for further diagnostics and can help estimating treatment costs for the owner. The modified Vet BLUE® protocol was selected as a structured lung-focused examination that provides clinically relevant information while remaining feasible in an emergency setting. In contrast, the VetFAST-ABCDE protocol encompasses evaluation of multiple organ systems, including cardiac and abdominal assessment as well as optic nerve sheath diameter measurement, and therefore represents a more comprehensive approach. Lung-focused ultrasound protocols, such as Vet BLUE® (5) are designed as rapid thoracic screening examinations rather than comprehensive multi-organ assessments. In emergency patients with respiratory distress, a balance must be achieved between diagnostic thoroughness and minimal patient handling. The present study deliberately evaluated a focused thoracic protocol to reflect real-world clinical decision-making, where rapid categorization of the primary intrathoracic pathology is prioritized.

Although Vet BLUE® is generally considered a rapid bedside examination, formal time measurements were not recorded in this study. Reported examination times in the literature vary depending on protocol complexity, operator experience, and patient cooperation (13, 14). A standardized protocol may facilitate a structured diagnostic workflow; however, examination duration ultimately depends on clinical stability, operator experience, and practical preparation requirements. While this focused approach offers practical advantages in the emergency setting, it also entails inherent limitations. The modified Vet BLUE® protocol applied in this study represents a focused sampling approach rather than a complete thoracic ultrasound examination. Evaluation of four acoustic windows per hemithorax, although standardized and clinically practical, does not allow systematic assessment of the entire lung surface. Consequently, focal, small, or centrally located lesions outside the predefined scanning zones may have remained undetected. This sampling-related limitation represents a potential source of diagnostic bias and should be considered when interpreting discordant findings between LUS and TXR.

A human study reported a significant reduction in the number of radiographs and computed tomography scans performed, following the routine implementation of LUS in emergency setting – without any negative impact on patient mortality – highlighting ultrasound as a safe and cost-effective alternative (15). One study in small animal medicine concluded, that a detection of ≥ 3 B-Lines in one scanned lung field is highly suggestive for alveolar-interstitial syndrome with an accuracy of 79% compared to CT, however the LUS is not recommended to be used as the sole imaging method (4). Although lung ultrasound has reduced reliance on radiographic imaging in human emergency medicine, translation to veterinary practice may require structured training programs, institutional protocols, and further evaluation of cost–benefit and workflow implications. In patients with repeated examinations to reassess or monitor progress of recovery, LUS has the clear advantage of being radiation free.

The present study was designed to compare imaging-based suspected diagnostic categories rather than to analyze individual ultrasound parameters (such as B-line counts, shred or tissue signs, or regional agreement between modalities), as reported in some previous studies. This decision was made to simplify analysis, and some aspects are quite subjective to assess. Studies about TXR in cats exemplify the complexity and non-uniformity of signs for CPE and broad range of equivocal vertebral heart score, which can make the diagnosis of congestive heart failure solely based on TXR difficult. Further, when there is fluid or severe edema, the cardiac silhouette can be obscured (16–18). In dogs, the radiographic signs are more uniform, and more sensitive cut-offs for vertebral heart score exist, but yet the limitation remains, that the cardiac silhouette needs to be clearly visible (19). A study from 2008, which compares quadrant spatial agreement between B-Lines on LUS and TXR for alveolar-interstitial syndrome finds fair agreement (*κ* = 0.24–0.56). The majority (61%) of all patients had CPE, but non-respiratory causes (e.g., neurological) were also included. Agreement was better in the caudal quadrants and improved when larger regions were compared. The severity of change for being classified positive varied between LUS and TXR, which might be the reason for such differences. The study group conclude that a pattern-based approach may provide more diagnostic usefulness (2). Revealing heart failure as primary cause for respiratory distress reached a sensitivity of 87% with LUS and 92.6% with TXR in our population. These numbers are similar to a recent study, in which they found a sensitivity of 84% for LUS and 85% for TXR for accurately detecting CPE (8). The limitation of this comparison is that in our study also animals with pleural effusion of cardiac origin are within this category, and we did not separately evaluated only CPE. With a similar working group, LUS was evaluated including a focused cardiac ultrasound to differentiate CPE from other causes of breathing difficulties in cats, increasing sensitivity to 97%, whereas specificity even reached 100% (3).

LUS demonstrated 100% sensitivity (95% CI, 74.1–100%) for detecting pleural effusion, compared to 81.8% sensitivity (95% CI, 52.3–97.9%) for TXR. This was also the only category in which LUS had a numerically higher sensitivity than TXR. Although the point estimate was higher for LUS, confidence intervals overlapped due to the limited number of cases. In contrast, another study detected pleural effusion with the Vet BLUE® protocol in only 2/3 (67%) cases, with the number of patients being very low (4). This finding might be explained, as we added an additional acoustic window, namely the xiphoidal view to detect free fluid.

Detecting masses can be challenging by LUS. In our study we were able to detect 4/7 (57%) neoplastic cases with the ultrasound. Depending on their location, for example centrally located or very small in size, other imaging modalities are necessary for diagnosis. Comparing LUS to CT, a previous study shows, that lung consolidation was correctly identified with the Vet BLUE® protocol in 14/24 (58%) and intrathoracic masses in 1/4 (25%) cases (4). With TXR we were able to detect 5/7 (71.4%) of all neoplastic cases. Due to higher spatial resolution and bypassing of overlapping tissue the CT also is superior to TXR. A study showed that 17/24 (81%) of pulmonary nodules were found on TXR, compared to the findings of the more advanced CT. Therefore, CT is recommended, especially in larger dogs (20).

In 14 patients, the initial suspicion of an intra-thoracic cause for the respiratory distress turned out to be incorrect after the first diagnostics. These animals had either an upper airway collapse with a stable heart disease, resolution of breathing difficulties after pain medication, neurological disorders or spontaneous resolution of the respiratory signs without any treatment. Exclusion of an intra-thoracic cause for respiratory distress was successful in 13/14 (92.9%) cases with TXR and 12/14 (85.7%) with LUS. The lung was the suspected category in all initially misdiagnosed patients, but further diagnostics showed that this assumption was wrong. The inclusion of patients in whom an intrathoracic cause of dyspnea was ultimately excluded reflects the clinical reality of emergency medicine. Initial clinical suspicion may overestimate the likelihood of respiratory pathology, particularly in animals presenting with nonspecific signs such as tachypnea or increased respiratory effort. The ability of imaging modalities to exclude intrathoracic disease is clinically relevant. Excluding such cases from analysis would have introduced spectrum bias and potentially overestimated diagnostic accuracy.

As discussed above, discordant cases were most commonly observed in pulmonary and neoplastic categories, where LUS may be limited by its inability to detect centrally located lesions not in contact with the pleural surface. Conversely, LUS demonstrated high sensitivity for pleural effusion, contributing to modality-specific strengths. To date, there are several studies comparing the accuracy of LUS and TXR in animals with respiratory distress, some only evaluating one imaging modality. As the exclusion criteria (e.g., trauma, effusion) differ and diagnostic accuracy is not always analyzed, everyone is encouraged to look closely before applying the results to one’s own patients. The most likely differential diagnosis based on medical history, signalment and physical examination findings should always be considered.

Concerning the results of the physical examination, in our study a heart murmur was the only parameter reaching a statistically significant effect for a cardiac disease being the cause for the symptoms in dogs, but not in cats. The four dogs of our study that were in congestive heart failure, but did not have a detectable murmur, was one with pericardial effusion, and the other three suffered of systolic dysfunction. In concordance to the present study, another working group showed a decrease of percentage of a heart murmur in cats in heart failure when compared to cats in a preclinical stage of the disease (21). The species difference observed in our study may be explained by known differences in cardiac disease pathophysiology. In dogs, particularly small breeds, myxomatous mitral valve disease is common and frequently associated with a clearly audible systolic murmur in cases of congestive heart failure. Some cats with cardiomyopathy may not exhibit audible murmurs in the absence of left ventricular outflow tract obstruction or systolic anterior motion of the mitral valve. These physiological differences may explain why auscultation was significantly associated with cardiac-related dyspnea in dogs but not in cats. Auscultation in dyspneic patients is inherently limited by respiratory noise, stress, and patient cooperation. Therefore, the presence or absence of a heart murmur should be interpreted cautiously and cannot reliably exclude or confirm a cardiac cause of dyspnea. In this study, auscultation findings were analyzed as exploratory clinical variables rather than as diagnostic determinants.

No other clinical parameter reached statistically significant correlation. By contrast, one study indicates that if cats show hypothermia, tachycardia, gallop sounds and profound tachypnea, a cardiac cause for respiratory distress is more likely (22). This could not be shown in another study testing if higher heart rates, lower rectal temperature and louder heart murmurs improved diagnostic accuracy of CPE in dogs and cats (8). Severity of the pathology and stress can also lead to significant tachycardia, as well as hyperthermia and an increase in respiratory rate. Taking detailed medical history, including duration and progression of clinical signs, known diseases, previous and current medications and considering the breed and age group of the patient, can help the emergency vet creating the list of differential diagnoses.

We chose respiratory distress as inclusion criterion, because other respiratory signs, like cough, usually do not require such an immediate action and are more commonly associated with an airway disease or an enlarged heart rather than with CPE (23). A previous study showed that certain respiratory parameters can give hints about the underlying pathology although they are not very specific, but the respiratory rate was not taken into account (24). Further, a different working group showed poor interclinician agreement of the interpretation of most respiratory clinical signs (25). Sedation was administered in a subset of patients to facilitate safe and adequate image acquisition. Sedation may reduce respiratory motion and improve image quality; however, it may also alter physiological parameters. As sedation was applied based on clinical necessity rather than a standardized protocol, it represents a potential confounding factor. Due to the limited number of sedated patients, subgroup analysis was not statistically feasible. Nevertheless, this approach reflects routine emergency practice and therefore enhances external validity.

Considering the limitations of the study, although the sample size is rather large for a veterinary study (*n* = 144), uneven distribution of diagnostic categories, particularly the small number of neoplastic cases, limited statistical power for subgroup-specific comparisons. As this study included consecutive dyspneic emergency patients, the case distribution reflects clinical reality rather than predefined sampling. Prior medical treatment, particularly administration of diuretics or oxygen therapy before imaging, may have altered radiographic and ultrasonographic findings and thereby influenced diagnostic performance. Although exclusion of pre-treated patients could theoretically reduce this confounding effect, such an approach would have limited the real-world applicability of the study and substantially reduced sample size in certain subgroups.

Although we did not exclude pathologies like pneumothorax or diaphragmatic hernia, which also would cause labored breathing, they did not occur in our patient population. Auscultation in some patients was not possible due to severely increased respiratory effort, non-stoppable panting or highly intensified lung sounds. Further, respiratory distress leads to limited information in some patients.

A potential source of bias in this study is the heterogeneity in operator experience. Examinations were performed by emergency veterinarians with varying levels of clinical experience, which may have influenced both image acquisition and interpretation, particularly for lung ultrasound. Ultrasound is inherently operator-dependent, and variability in technical skills and pattern recognition may affect diagnostic performance. However, thoracic radiography interpretation is also subject to observer variability. While this heterogeneity represents a limitation, it increases the external validity of the study, as it reflects real-world emergency practice rather than a controlled expert-only setting.

The discordant cases in which TXR correctly classified the disease category while LUS did not likely reflect intrinsic limitations of lung ultrasound. LUS primarily assesses pleural surface abnormalities and may fail to detect centrally located lesions or other pathologies not in contact with the pleura. The modified Vet BLUE® protocol applied in this study represents a focused four-point-per-hemithorax sampling approach rather than a comprehensive thoracic ultrasound examination. Consequently, not all lung regions were systematically assessed, and certain pathologies may have remained undetected if located outside the predefined acoustic windows. These limitations reflect the inherent trade-off between rapid bedside applicability and comprehensive thoracic evaluation and should be considered when interpreting discordant findings.

The time between LUS and TXR was kept to a minimum, but there were still some animals which needed a pause and oxygen supplementation between the imaging modalities to ensure the best patient care. To reduce the effect of time, the allowed maximum time between examinations was limited to 1 h. Nevertheless, it cannot be ruled out with certainty that slight changes during this time may have led to disagreement.

We performed the LUS with a microconvex probe, as it is widely used in veterinary medicine in emergency settings. Further, the depth can be increased more than with a linear probe which eliminates the need to change transducer when a xiphoidal view is performed. A study group showed equal performance of the microconvex and the linear transducer in 200 dogs and cats with dyspnea in identifying B-lines and diagnosing CPE, pneumonia and lung neoplasia (26). This finding is in contrast to another study in which they show superiority of detecting and correctly classifying hyperechoic vertical artifacts of the linear transducer, compared to the microconvex one in eight dogs without signs of respiratory distress (11). Both mentioned studies agree that the performance of a phased array transducer is inferior. Further, they are in accordance that the performance depends on the experience of the observer, the ultrasound machine and the settings of the preset used. LUS must be assessed in combination with the clinical and laboratory findings, and there is a higher diagnostic value if more than just B-lines are assessed (8, 27). This finding is supported by another working group, who conclude that B-lines or comet-tail artefacts and free fluid are not specific, but nodular lesion and consolidations in context with clinical findings have shown a trend towards neoplasia or pneumonia (27).

Although there are benchmarks and proposed patterns for some diseases (5), this is not always in agreement in every case, and each patient has to be evaluated individually, considering the most likely differential diagnosis. For instance, in our study some patients with severe CPE showed shred signs, which is in contrast to another study in dogs with cough, where shred signs did not occur in this category of patients (9).

A standardized focused cardiac ultrasound examination was not mandatory in this study. While integration of FOCUS may improve diagnostic accuracy, particularly in differentiating cardiogenic from non-cardiogenic causes of respiratory distress, the present study was designed to reflect real-world emergency workflow. It has been shown that with 6-h training quite inexperienced interns or residents are able to improve their skills on focused echo examination in the emergency room and detect fluids and atrial enlargement, but are not able to carry out a detailed assessment (28). Future studies incorporating systematic cardiac point-of-care ultrasound may further refine diagnostic performance.

As the underlying causes varied, no standardized workup was performed. It was at the discretion of the specialist to decide which tests and treatments should be carried out. Still, it could not be completely ruled out that there was not only one cause for breathing difficulties, as there might be more than one underlying disease in some patients. This is consistent with another working group that examined radiological signs in cats with pleural effusion in order to predict the etiology of the disease (18).

In animals with severe respiratory distress, it is particularly challenging to obtain TXR with sufficient quality, as positioning may not be well tolerated, and high breathing rates increase the chance of blurry images. LUS can help to achieve higher diagnostic certainty in patients when the TXR is suboptimal or shows results that can be interpreted in different ways. In one study, CPE was misdiagnosed in 16/88 (18%) dogs with cough when the images were not of sufficient quality (poor positioning or expiratory pictures) (9).

The different performance in the blinded re-evaluation may have been due to several reasons: First, the primary emergency vet was not blinded to any findings of the two imaging modalities or clinical findings to assure best patient care. This could have influenced the assessment, although we encouraged all of them to evaluate each diagnostic modality on its own. For the blinded analysis, only a few details were given, and only one imaging modality at a time was reviewed. For the emergency vet and the ECC-resident, who evaluated both imaging modalities, the cases were set a different order to avoid bias of remembering some details from certain patients. In LUS as well as in TXR, the experienced reviewers noted that the image quality was only acceptable in some animals, and especially with LUS they would have added more acoustic windows and evaluated the heart in every single patient. But as the videos were generated by vets with 1–4 years of experience in the emergency service, this was not possible in every case. Proper ultrasound machine settings are extremely important for LUS. Human critical care literature recommends specific machine settings [e.g., focal zone at the pleural line, harmonic imaging off, low frequency, which may influence artifact appearance and interpretation (29)]. To the best of the authors’ knowledge, no such review or consensus statement exists in veterinary medicine yet.

Further, some B-line artefacts may have been misclassified by inexperienced examiners, as there are various hyperechoic vertical artefacts (e.g., Z-lines) that can only be distinguished by adjusting the ultrasound settings and with more expertise in interpreting LUS. TXR is easier to perform when the animal allows correct positioning, for then two images are sufficient. As there were several videos needed for the LUS, the possibility that a sequence showed reduced quality was more likely. Additionally, the initial therapy from referring vets was on the information sheet for the reviewers. As this was not always correct, it could have been misleading in some cases. The numbers from the blinded re-evaluation probably show a better picture of how well the modalities, when used on their own, lead to a correct diagnosis. The observed differences in interobserver agreement may reflect variations in interpretative strategy rather than differences in competence: Less experienced clinicians may rely predominantly on conspicuous radiographic patterns, potentially leading to higher categorical agreement. More experienced observers may incorporate subtle findings and a broader range of differential considerations, potentially increasing categorical variability. No formal calibration session was conducted prior to blinded re-evaluation, as the study aimed to reflect real-world variability in emergency imaging interpretation. This lack of calibration may represent a limitation. Based on this aspect, comparing our study to others is limited, as most of them do not test interobserver agreement on suspected diagnosis. One study evaluated the agreement of number of B-lines, and they achieved a higher kappa (*κ* = 0.86) (8).

Further studies are warranted to see if a more detailed protocol and categorization may lead to a better performance of those primary imaging modalities or how an implementation of a point-of-care ultrasound of the heart or laboratory results, such as cardiac biomarkers, improve diagnostic accuracy. It also highlights the effect of examiner experience on diagnostic performance of an imaging modality and a more standardized approach with defined criteria for experience should be aimed for.

## Conclusion

This study shows that LUS, using a modified Vet BLUE® protocol, should not be seen as a replacement, but rather as a complement when it comes to diagnosing patients with respiratory distress. A thorough examination of an expert and re-evaluation when the clinical signs improved are essential, especially when the suspected diagnosis is not concluded with a high degree of certainty. Although this newer diagnostic tool has its benefits, TXR has been shown to be superior in total across all categories in the used setting. Therefore, TXR should still be used when a clear diagnosis cannot be found on ultrasound and to get a more complete overview of the whole thorax. As the inclusion of evaluating the heart with ultrasound improved the performance, it is recommended to integrate a point-of-care ultrasound of the heart in every patient.

## Data Availability

The datasets generated and/or analyzed during the current study are available from the corresponding author on reasonable request.
